# Synthesis of Artemether-Loaded Albumin Nanoparticles and Measurement of Their Anti-Cancer Effects

**DOI:** 10.3390/biomedicines10112713

**Published:** 2022-10-26

**Authors:** Zeynab Pirali-Hamedani, Ardeshir Abbasi, Zuhair Mohammad Hassan

**Affiliations:** Department of Immunology, Faculty of Medical Sciences, Tarbiat Modares University, Tehran 1411713116, Iran

**Keywords:** colorectal cancer, artemether, albumin–artemether nanoparticles

## Abstract

Colorectal cancer is the third most common cancer in the world. Due to the side effects of common treatments such as chemotherapy and radiotherapy, the use of herbal medicines has received much attention. Artemether (ARM) is an herbal medicine derived from artemisinin, which has many anti-tumor properties. However, factors such as low solubility and short half-life have limited the use of artemether in clinical practice. In this study, we aimed to reduce these limitations by encapsulating artemether in human serum albumin (HSA). The hydrodynamic diameter and the zeta potential value of ARM-ALB nanoparticles (NPs) were 171.3 ± 5.88 nm and −19.1 ± 0.82 mV, respectively. Comparison of the effect of free and encapsulated artemether on CT 26 cell line showed that the use of artemether in capsulated form can reduce the effective concentration of the drug. Additionally, in vivo studies have also shown that albumin–artemether nanoparticles can control tumor growth by increasing the production of cytokine IFN-γ and decreasing the production of IL4. Therefore, ARM-ALB nanoparticles have greater anti-tumor effects than free artemether.

## 1. Introduction

Colon cancer (CLC) is the third most common cancer in the world and accounts for approximately 10% of diagnosed cancers annually [1]. During recent decades, CRC has been recognized as the second most common malignancy in women and the third most common malignancy in men worldwide in terms of incidence [2]. The geographical distribution of CLC also varies, with the highest incidence occurring in developed countries [3]. The change of the normal colonic epithelium, including dysplasia and metaplasia to a cancerous tumor, both non-polyposis and polyposis, which occurs as a result of genetic changes and functional impact, can lead to colon cancer [4]. There are two types of risk factors that increase the risk of colon cancer: genetic factors and environmental risk factors. According to studies, a positive family history can play a role in colon cancer [5]. Meanwhile, factors such as drug use, processed meat, alcohol and red meat consumption, low consumption of vegetables and fruits, and body fat and obesity can elevate the risk of colon cancer [6]. Studies also show that other factors such as aspirin use, type 2 diabetes, male gender and inflammatory bowel disease are also risk factors associated with colon cancer [7]. Although there are various treatments such as surgery, chemotherapy, radiation therapy, immunotherapy and nutritional supplements for this cancer, due to the side effects seen in all these treatments, the success rate of treating this cancer is not encouraging. For example, chemotherapy, although it is a common treatment for cancer, due to its non-selective actions, requires higher doses and therefore leads to drug resistance and causes severe toxicity to normal cells [8,9,10,11]. Moreover, these treatments can cause complications such as nausea, anorexia and numbness in patients and overshadow their quality of life.

In recent years, herbal medicines have gained much attention due to their low toxicity and fewer side effects [12]. Artemisinin, which was first discovered in 1972 by a Chinese scientist named You you tu, is an anti-malarial herbal medicine that was first isolated from Artemisia Annua, an herb employed in traditional Chinese medicine [13]. Studies show that artemisinin and its derivatives also have many anti-tumor properties [14,15,16]. The structure of artemisinins belongs to sesquiterpene lactones, which have an unusual endoperoxide bridge in their structure. These endoperoxide bridges can react with iron and lead to the production of free radicals. Because tumor cells contain more intracellular iron than normal cells, it has been shown that artemisinin and its derivatives can selectively induce apoptosis in cancer cells [17]. Artemisinin and its derivatives exert their antitumor effects through mechanisms such as regulating immune system activity, inhibiting tumor angiogenesis, preventing invasion and metastasis, and inducing apoptosis in tumor cells [18,19,20,21]. In 2015, Michaelsen et al. showed that artemisinin derivatives could reduce the risk of advanced metastasis in prostate cancer [22].

Artemether (ARM) is the methyl ethylene derivative of artemisinin [23]. Studies show that artemether can effectively inhibit the growth of a variety of tumors. In 2009, Zhi-Ping Wu found that artemether specifically inhibits the growth of cerebral glioma by penetrating the blood–brain barrier and inhibiting angiogenesis [20]. In 2016, studies on breast carcinoma revealed that artemether inhibited the growth of MCF7 cells. In 2016, studies on breast carcinoma by Samandari-Bahraseman MR et al. demonstrated that artemether inhibited the growth of MCF7 cells [24]. Moreover, the use of artemether with Vincristine and Doxorubicin, compared to the use of either alone, reflects a significantly higher cytotoxic effect. Although artemether has many anti-tumor properties, it can pose challenges for clinical use. For example, artemether’s short half-life limits its clinical use. It was found, in earlier research, that artemether can have a maximum half-life of 8 h if injected intramuscularly under optimal conditions [25]. Factors such as low solubility and low bioavailability also limit the clinical use of artemether [26]. Although many and successful studies have been conducted on the antitumor effects of artemether, some research has shown that artemether can increase irregular Ca^2+^ transients during pacing and spontaneous Ca^2+^ events during rest periods and causing prolonged action potentials (AP) and nervous system disorders.

In a study conducted in 2019 by Ana Carolina Moreira Souza and colleagues, the mechanism of artemether toxicity on single cardiomyocytes and the protective effect of nanoencapsulation were investigated. The results of this study showed that the use of artemether in the form of nanocapsules prevented these adverse effects and that nanocapsules can be used as a suitable alternative to artemether alone especially to treat malaria [27].

One way to increase the effectiveness of drugs is to use drug carriers. Drug carriers mitigate side effects by tumor-specific targeting and utilizing the enhanced permeability and retention (EPR) effect [28]. Albumin is a protein carrier widely used for drug targeting. HSA is a three-domain allosteric macromolecule that is composed of 65–68% α-helix with multiple turns and little β-sheet content (~1–3%); and it is also the most abundant plasma protein (50–35 g/L) in human serum with a molecular weight of 66.5 kDa [29]. This protein, like most plasma proteins, is made in the liver [30]. Human serum albumin has a half-life of 19 days and is a highly soluble acidic protein. In addition, biodegradability, non-toxicity, non-immunogenicity, appropriate size and long-term circulation are factors that make it an effective drug carrier [31,32]. Albumin plays an important role in the transport of many endogenous and exogenous ligands (such as hormones, FA, nitric oxide, bilirubin, metalloporphyrins, warfarin, aspirin, phenylbutazone, etc.) in the bloodstream and extravascular spaces [33,34]. Binding chemotherapeutic drugs to albumin and the delivery of drugs by albumin can significantly affect their efficacy. Moreover, albumin can bind to many endogenous ligands, such as physiologically significant fatty acids that can affect metabolism and tumor proliferation [35]. HSA through non-covalent interactions can bind to various drugs and peptide compounds. Reactive groups on the nanoparticle surface, such as carboxyl, amino, and thiol, facilitate covalent ligand binding and surface modification. HSA can be used to load a variety of drugs for delivery via the circulatory system because it has excellent ligand-binding properties. HSA not only has high binding affinity sites for the loading of therapeutic drugs, but also it has high stability. HSA has the capacity to bind to seven long-chain fatty acids at several binding sites with different affinities [36].

Because cancer cells consume a lot of nutrients and energy, they overexpress nutrient transporters such as albumin-binding proteins (ABPs). One of the important reasons for using albumin as an anti-tumor drug carrier is that albumin-binding proteins such as gp60 and SPARC are abundantly expressed in tumor cells. For this reason, ABPs can act as a target for drugs with albumin coating and cause more drug uptake by tumor cells [37,38,39]. Albumin also has functional groups such as amino and carboxylic groups that can be used to functionalize albumin nanoparticles with target ligands or active drugs. In addition, the stability of albumin-based nanoparticles allows the systematic delivery of various agents without degradation [10,40]. In previous studies, it has been shown that the preparation of albumin–artemether NPs through the desolvation method has led to the achievement of nanoparticles with advanced solubility properties and, as a result, increased drug efficiency [41,42].

In this study, we intended to reduce the limitations of artemether and increase its effectiveness by encapsulating artemether with albumin. For this purpose, albumin nanoparticles were prepared by the desolvation method, and their effect on mouse colon cancer was investigated by in vitro and in vivo studies.

## 2. Material and Methods

### 2.1. Materials

CT26 colon cancer cell line was obtained from the department of immunology, faculty of medical sciences, Tarbiat Modares University. The trypsin-EDTA (0.25%) was purchased from Bio IDEA (Tehran, Iran). The Glutaraldehyde (cross linker) 25% solution, human serum albumin (HSA, >98.0% pure), 3-(4, 5dimethythiazol-2-yl)-2, 5-diphenyl tetrazolium bromide (MTT, >98.0% pure), and Ethanol 96% were acquired from Sigma-Aldrich (St. Louis, MO, USA). The cell culture medium (RPMI), antibiotic (penicillinstreptomycin) and fetal bovine serum (FBS) were provided by Gibreast canceroBRL (Life Technologies, Paisley, Scotland). We used artemether powder (CAS no: 71963-77-4, >98.0% pure) (Sigma Aldrich, St. Louis, MO, USA). The annexin-V apoptosis kit was acquired from MabTag (Friesoythe, Germany).

### 2.2. Preparation of ARM-HSA Nanoparticles

In order to prepare ARM-HSA nanoparticle, 20 mg of Artemether was firstly added to 4 mL ethanol. Then, 200 mg of HSA was dissolved in 1.0 mL of Milli-Q water. The HSA solution was placed on a stirrer with a constant stirring rate of 500 rpm at room temperature and ARM solution was added dropwise (1.0 mL/min). After 2 h, 30 µL of Glutaraldehyde solution (25%) was added as a cross-linker. The solution was then stirred for 24 h. After 24 h, 6 consecutive centrifugations (15 min, 30,000× *g*, at 4 °C) were performed to purify the nanoparticles. At each stage, the pellet was re-suspended in pure water using an ultrasonic bath (Wised WUC-D10H) for 5 min. Finally, ARM-HSA nanoparticles were transferred to a freeze dryer (Zirbus Vaco 5, Zirbus Technology, Bad Grund, Germany) for 24 h at −50 °C after having been pre-frozen for 24 h at −70 °C.

### 2.3. Investigation of Properties and Structure of Nanoparticles

#### 2.3.1. FTIR (Fourier-Transform Infra-Red) Spectroscopy

FTIR determine the functional groups present in the chemical structure, and can estimate the type of bonding between the drug molecule and the materials/polymers used to prepare the nanoparticles/carrier and the structural changes that result from chemical reactions from the available spectra. In this study, this technique was used to investigate the structure of NPs. For this purpose, 100 mg of potassium bromide was added to each 2 mg of sample and the absorption spectra of resulted pellets were recorded at 25 °C, in a range of 400–4000 cm^−1^ at 1 cm^−1^ resolution by FTIR spectrophotometer (PerkinElmer Frontier, Waltham, MA, USA).

#### 2.3.2. Investigation of ARM-HSA NPs Morphology by Scanning Electron Microscopy (SEM) and Transmission Electron Microscopy (TEM)

*Scanning electron microscope* (SEM) was used to investigate the morphology and microstructure of nanoparticles and compare the appearance of ARM-HSA NPs with free artemether. The lyophilized nanoparticles were coated with gold sputter, and analyzed under SEM (FEI-Nova NanoSEM 450, Thermo Fisher, Waltham, MA, USA). For the TEM method, the sample was diluted with distilled water and dried at room temperature by placing it on a carbon-coated copper grid. The dried sample was observed under the TEM (PHILIPS CM300, PHILIPS, Cambridge, MA, USA, and 200 kV). At the end, the diameter, morphology and microstructure of nanoparticles were evaluated by using Image analyzer (Digital Micrograph software 1.81.78, Gatan, Pleasanton, CA, USA).

#### 2.3.3. Investigation of Particle Size, Polydispersity Index and Zeta Potential with DLS

The DLS method was used to more accurately evaluate the ARM-has NPs size. Unlike SEM analysis, which uses the powder form of nanoparticles, DLS analysis is performed in a liquid medium and due to the reflection of particles in the liquid medium, the hydrodynamic size of the particles is obtained. Because nanoparticles are ultimately used in the liquid system rather than in powder form, the particle size in the DLS analysis is closer to the actual size of the nanoparticles, which is why nanoparticle size evaluation with DLS is important [43]. A total of 1.5 mL of deionized water was added to the samples and after the samples were suspended, their size, polydispersity index (PDI) and zeta potential were recorded by particle size analyzers (Malvern Instruments Ltd., Malvern, UK).

#### 2.3.4. Process Yield and Drug Loading

First, different concentrations of artemether were prepared and their absorption was measured with a spectrophotometer at 212 nm [44]. A curve was plotted as the standard curve. During the initial stages of nanoparticle preparation, after each centrifugation, the supernatant was removed and collected. Then amount of free ARM absorption was measured by UV spectrophotometry in the range of 200–300 nm [45]. Using the standard curve, the concentration of free artemether was obtained. Then, the amount of encapsulated ARM in the HSA was determined by subtracting the amount of free ARM from the total used ARM. Finally, the following equations were used to calculate process yield (1) and DL (%) (2), respectively [46]. UV-Vis analyses were accomplished at 25 °C by UV spectrophotometer (OPTIZEN 3220UV, Korea).

(1) $$\;\;process\;yield\;\left(\%\right)=\frac{Amount\;of\;total\;ART\cdot HSA\;NPs}{Total\;amount\;of\;ART\;+\;total\;amount\;of\;HSA}\;\;\times 100$$

(2) $$DL\left(\%\right)=\frac{Amount\;of\;total\;used\;ART-Amount\;of\;free\;ART\;}{Total\;amount\;of\;ART\cdot HSA\;NPs}\times 100$$

#### 2.3.5. Solubility Evaluation

To compare the solubility of nanoparticles with free artemether, the nanoparticles and free Artemether were weighed so that each of them contained 1 mg of artemether. (The amount of drug is the same in both forms: 1 mg Artemether in free form and 1 mg Artemether in capsule form.) Then, 1.0 mL of deionized water was added to them separately and they were stirred for 10 min. They were then centrifuged (10,000× *g*) for 15 min to precipitate undissolved nanoparticles and ARM. Artemether level was detected in supernatant using UV spectroscopy at 212 nm.

#### 2.3.6. Drug Release Evaluation

Phosphate buffer (pH = 7.4) was used to simulate the physiological condition and sodium citrate buffer (pH = 5.5) was used to simulate the tumor microenvironment. drug release from NPs was investigated according to the previously published article [47]. Accordingly, a certain amount of ARM-HSA NPs was placed separately in several of Eppendorf tubes containing buffers. The tubes were placed in a shaker incubator (100 rpm) at 37 °C. After specified time intervals (0, 4, 8, 12, 16, 20, 24, 36, 48, 72 h), each tube was centrifuged at 7000 rpm for 5 min to precipitate the released artemether from the NPs. Then, 1.0 mL of ethanol was added to the precipitated artemether and the amount of released ARM was measured using the UV spectrophotometer at 212 nm. The concentration of the released ARM was obtained using the standard curve. To avoid the interference of artemether and albumin absorbance peaks in release examination, there was sample containing free nano albumin (without artemether) as a control in each step.

### 2.4. In Vitro Studies

#### 2.4.1. Cell Culture

The CT26 mouse colon cancer cell line was obtained from the Pasture Institute, Cell Bank of Iran (NCBI, Tehran, Iran). Cells were cultured in RPMI containing 10% FBS and 1% antibiotic (penicillin 100 U/mL, streptomycin 100 μg/mL). The cells were incubated at 37 °C in a humidified 5% CO_2_ atmosphere (incubator; Binder, BIND_6021, Claydon, UK).

#### 2.4.2. MTT Assay

To evaluate the anti-proliferation activity of nanoparticles and compare it with the effect of free artemether, MTT test was performed.

Ct26 cells were seeded into the wells of a 96-well plate at a density of 6000, 7000, 8000 cells/well for 24, 48, 72 h evaluation, respectively. MNCs isolated from the spleen of BALB/c mice were seeded as normal cells at a density of 1 × 10^5^ cells/well. After 24 h of incubation, culture medium was replaced with a fresh medium. Then, cells were treated with various concentrations of artemether and an equivalent amount of ARM in nanoparticle formulation (0, 5, 15, 30 and 50 μg/mL) for 24, 48 and 72 h incubation periods. After incubation, the medium was removed and the cells were exposed to 100 µL of MTT solution (0.5 mg/mL) for 4 h at 37 °C. Afterwards, MTT solution was removed and 100 µL of DMSO was added to each well to dissolve the Formazan crystals. The absorbance was measured with an ELISA reader at 450 nm. All the aforementioned steps were performed for normal cells and cancer cells similarly.

#### 2.4.3. Apoptosis Assay

Annexin-V assay, based on cell membrane changes caused by apoptotic processes, is a widely used method for apoptotic analysis as well as discrimination between necrosis and apoptotic cell death [48]. For this purpose, CT26 cells and MNCs were seeded in a 12-well culture plate at a density of 1 × 10^5^ and 10^6^, respectively, and incubated for 24 h at 37 °C. After 24 h, cells were treated by an effective dose of ARM (50 µg/mL) and equivalent amount of ARM in nanoparticle formulation for 24 h. After incubation, cells were trypsinized and centrifuged (1500× *g*, 15 min at 4 °C) and the pellet was re-suspended in Annexin-V binding buffer. Then, 5 µL of Annexin-V FITC conjugate and 5 µL of PI (propidium iodide) were added to cell suspension and analyzed by flow cytometry.

### 2.5. In Vivo Studies

#### 2.5.1. Tumorization of BALB/c Mice

Cytokine production of the immune cells was measured by ELISA. In the first phase, 25 mice were purchased from the Royan Institute. Each mouse was injected subcutaneously with 100 μL of PBS containing 600,000 CT26 cells. After 2 weeks, the tumors were clearly visible. The ethical approval code for this research is IR.MODARES.REC.1398.161.

#### 2.5.2. Treatment of Mice with Drugs

When the tumor size of the mice reached 200 mm^3^, treatment of the mice began. In the second stage, mice were divided into 5 groups (6 mice in each group) receiving free artemether, ARM-HSA NPs, Cyclophosphamide, albumin and PBS as a control group. Drugs were injected into tumor mice in 7 steps. At each injection stage, free and encapsulated artemether (both at a concentration of 10 mg/kg of artemether) were injected intramuscularly into each mouse [49]. PBS solution containing 5% ethanol was used to dissolve free artemether and PBS was used to dissolve nanoparticles. Tumor volume measurements were started one day before the first injection and was repeated every other day. After the drug injection steps, three mice from each group were kept for survival analysis. The tumor size of the mice was measured every other day for up to 60 days. For ethical reasons, mice with a tumor size of 2000 mm^3^ and above were considered dead, and mice with a tumor size of less than 2000 mm^3^ were considered alive.

#### 2.5.3. Isolation and Culture of MNCs

Two mice from each group were randomly selected to measure cytokine levels. The mice were anesthetized using ketamine and xylazine and their spleens were removed, and then the mice were sacrificed and euthanized. After spleen isolation, the Splenocytes of each mouse were isolated by perfusion and counted. The red blood cells were removed using a lysis buffer, and finally, the MNCs were seeded at 1 million cells per well in a 24 well culture plate. The cells were activated with 25 μg/mL of tumor cell lysate for 72 h. Supernatant fluid obtained on day 4 was stored frozen at −70 °C until being analyzed for IFN-g and IL4 levels by ELISA.

#### 2.5.4. ELISA

ELISA plate wells (Costar 3590; Costar, Cambridge, MA, USA) were incubated overnight with 100 μL of the diluted capture antibody. The wells were blocked with 300 μL of PBS containing 1% bovine serum albumin for 1 h. After three washing steps by PBS containing 0.05% tween 20. A total of 100 μL of standard diluted cytokine series and test samples were added and the plate was incubated for 2 h. The fluid from the wells was aspirated and the plates were washed. Then, 100 μL of detection antibody was added to each well and incubated for 2 h. Subsequently, the wells were washed and then 100 μL of streptavidin-HRP was added to each well and incubated for 20 min. Aspiration and wash steps were repeated and then 100 μL of TMB was added as a substrate. Finally, after 20 min of incubation, 50 μL of sulfuric acid was added as a stop solution and the plates were read at 450 nm.

### 2.6. Statistical Analysis

Statistical significance was evaluated using *t*-test was used for two-group comparison and two-way ANOVA for multiple-group analysis by using Graph Pad Prism 7.0 for Windows (Graph Pad Software, Inc., San Diego, CA, USA). * *p* < 0.05, ** *p* < 0.01, *** *p* < 0.001 and **** *p* < 0.0001 were considered as significant levels for all analyses performed. Data were presented as mean ± standard deviation in triplicate experiments. The flow cytometry results were analyzed by flowing software version 2.4.1.

## 3. Results

### 3.1. Characterization of Artemether–HSA Nanoparticles

Nanoparticles were characterized by Fourier transform infrared spectroscopy (FTIR), Dynamic light scattering (DLS) and scanning electron microscope (SEM) techniques, and after confirming the standard features, they were used.

#### 3.1.1. Fourier Transforms Infrared (FTIR) Spectroscopy

**Figure 1:**
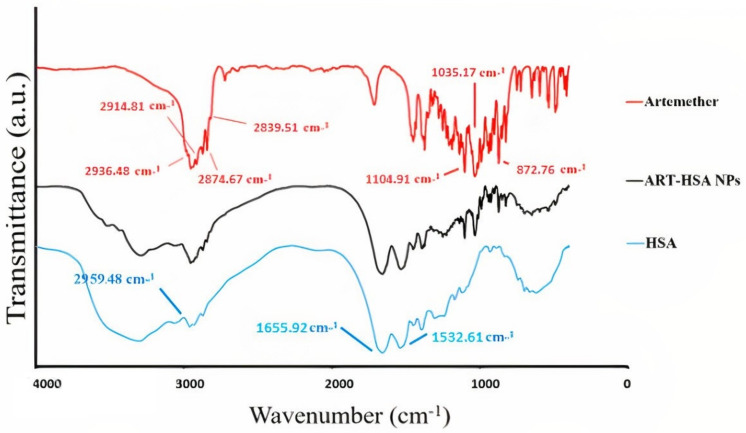
The FTIR results of artemether, albumin and ARM-Alb NPs. The presence of the main peaks of artemether and albumin in the nanoparticle structure indicates that their structure remains intact after the encapsulation process.

The main structure and bonds of synthesized nanoparticles were confirmed by FTIR analysis (Figure 1). FTIR spectrum of free ARM showed three main peaks at 1035.17 cm^−1^ (C-O), 1104.91 cm^−1^ (C-O-C) and 872.76 cm^−1^. The spectrum of human serum demonstrated two characteristic peaks at 1655.92cm^−1^ and 2959.48 cm^−1^ related to amide I and amide II bonds. All the above-mentioned absorption bands were also observed in artemether–HSA NPs which indicated no chemical modifications in artemether and albumin structure during the encapsulation process. Although the small adsorption peak of artemether in nanoparticles indicates a small amount of un-encapsulated artemether, the significant reduction in the artemether adsorption spectrum in the nanoparticle diagram proves its success in artemether encapsulation process.

#### 3.1.2. Zeta Potential, PDI and Size of Nanoparticles

**Figure 2:**
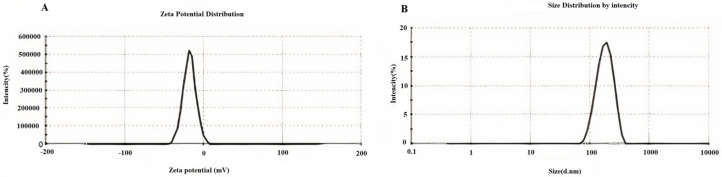
Zeta potential (**A**) and Dynamic light scattering (**B**) of albumin–artemether nanoparticles show the surface charge and particle size.

Zeta potential, PDI index, and mean nanoparticles size were determined through the DLS technique [50] (Table 1). According to previous studies, 100 to 200 nanometers is the optimal size for nanoparticles [51]. Table 1 shows that the nanoparticles in this study have a standard size (171.3 ± 5.88). PDI is an indicator of particle size dispersion. In 2018, a study on the effect of PDI on drug nano-carriers showed that the optimal PDI for albumin nanoparticles was about 0.2 or less. The PDI index in this study was 0.132 ± 0.006, which is in the acceptable range [52]. Diagrams of zeta potential and nanoparticle size are shown in Figure 2.

#### 3.1.3. Analysis of Nanoparticle Shape Using Electron Microscopy (SEM)

**Figure 3:**
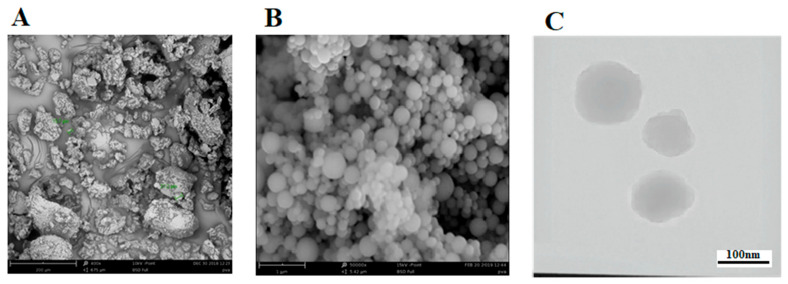
SEM Image of free artemether at 400× magnification (**A**). SEM Image of encapsulated artemether at a magnification of 50,000× (**B**). TEM image of ARM-HSA nanoparticles (lyophilized powder) (**C**). The conversion of the irregular shape of atemether into a regular, spherical shape confirms the encapsulation process.

SEM was used to perform the morphological characterization of the developed NPs (Figure 3). Figure 3A demonstrates the image of the free artemether in 400× magnification and Figure 3B shows the image of the encapsulated artemether (50,000× magnification). The free artemether seems to have an unorganized shape, while the nanoparticles are seen in a round shape with a smooth and regular surface. Additionally, the round shape of nanoparticles with a size of less than 200 nm was evidently confirmed by the TEM micrograph image (Figure 3C). Previous studies show that albumin nanoparticles that have a spherical shape and their size In the range of 50 to 300 nm, they are suitable for drug delivery [41,42].

#### 3.1.4. The Measure of Encapsulation Efficiency, Drug Loading and Process Yield

Using the spectrophotometer and standard artemether curve, the amount of drug loading (the ratio of the amount of artemether loaded to the total nanoparticles produced) was determined and the process yield and encapsulation efficacy were calculated. Drug loading in this study was found to be 5.1%. Encapsulation efficiency of NPs was 73.6% and the process yield was 65%.

#### 3.1.5. The Release and Solubility Profile of Nanoparticles

**Figure 4:**
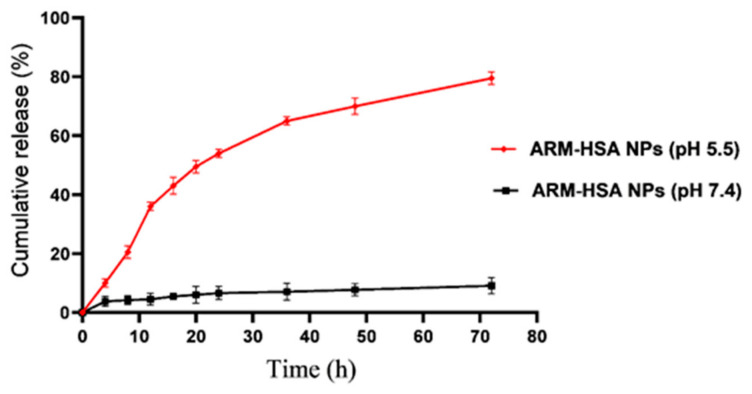
The release rate of nanoparticles exposed to citrate buffer with acidic pH and phosphate buffer with neutral pH at intervals of 0 to 72 h.

Drug release was monitored in a physiological-like environment (Phosphate buffer) and a cancer-like environment (citrate buffer) for 72 h (Figure 4). According to Figure 3, in 72 h, approximately 10% of the drug was released from the nanoparticles under similar physiological conditions. However, in the tumor environment, this amount shows a rise of about 75% in drug release rate. The result of dissolving the free and encapsulated artemether in deionized water showed that if the artemether is encapsulated with albumin, its solubility will increase up to 50 times.

### 3.2. MTT Assay

**Figure 5:**
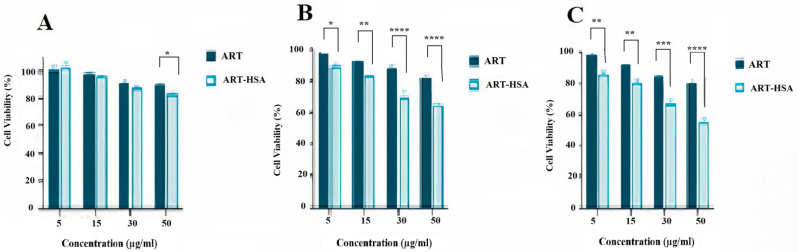
Results of artemether and nanoparticle cytotoxic effect on CT26 cell line in 24 (**A**), 48 (**B**), and 72 (**C**) h. Asterisks show the statistically significant difference between various treatments. (* *p* < 0.05, ** *p* < 0.01, *** *p* < 0.001, and **** *p* < 0.0001). Data are presented as mean ± SD.

**Figure 6:**
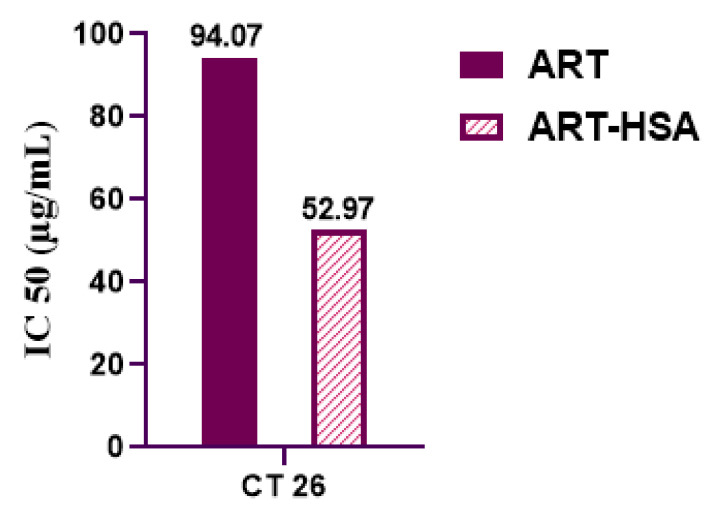
Comparison of the IC50 value of free artemether with encapsulated artemether.

MTT assay was performed for analyzing the in vitro cytotoxic effect of ALB-ART NPs on colon cancer cell line (CT26 cell line) (Figure 5). For this purpose, CT26 Cells were treated with various concentrations of artemether in the form of free and encapsulated artemether for 24, 48 and 72 h periods. As a control group, MNCs were treated with the same dose of drugs for the same period. The results of the MTT assay are shown in Figure 4. As observed, at 48 and 72 h, at all concentrations, the cytotoxicity effect of encapsulated artemether was significantly greater than the free artemether. Unlike the cytotoxicity effect of free artemether, which does not increase much after 48 h, the cytotoxicity effect of nanoparticles increased after 48 h, so that at the end of 72 h, cell survival decreased to about 50%. Moreover, the maximum effect of artemether at 72 h and in 50 μg/mL concentration was due to nanoparticle formulation which had a statistical difference with the free artemether (*p* < 0.0001). According to the results, the half-maximal inhibitory concentration (IC50) value of free and encapsulated artemether was calculated (Figure 6). The IC50 value was calculated as 52.97 μg/mL for nanoparticles and 94.07 μg/mL for free artemether.

### 3.3. Apoptosis Analysis

**Figure 7:**
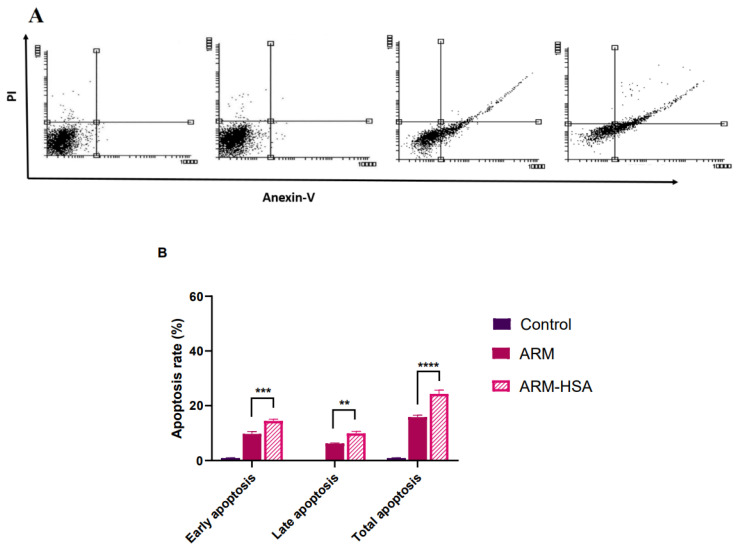
Results of Annexin-V FITC/PI assay after 24 h of drug treatment. Dot plot diagrams show apoptosis in the treatment groups (**A**). The bar chart shows early, late, and total apoptosis separately (**B**). Asterisks show the statistically significant difference between various treatments. (** *p* < 0.01, *** *p* < 0.001, and **** *p* < 0.0001). Data are presented as mean ± SD.

Cell apoptosis was analyzed by Annexin-V FITC/PI assay to accurately investigate the mechanism of cell death induced by artemether. CT26 cells and MNCs (as a control group) were treated with the 50 μg/mL concentration of artemether and an equal amount of artemether in nanoparticle formulation for 24 h. In the apoptosis assay analysis, evaluated cells are distinguished separately into early apoptotic, late apoptotic, necrotic, and live cells [53,54]. The results of apoptosis analysis are displayed in Figure 7. According to the figure, there is a significant difference between total apoptosis due to free artemether and total apoptosis due to encapsulated artemether (*p* < 0.0001).

### 3.4. Results of In Vivo Studies

#### 3.4.1. Effect of Drugs on Tumor Growth

**Figure 8:**
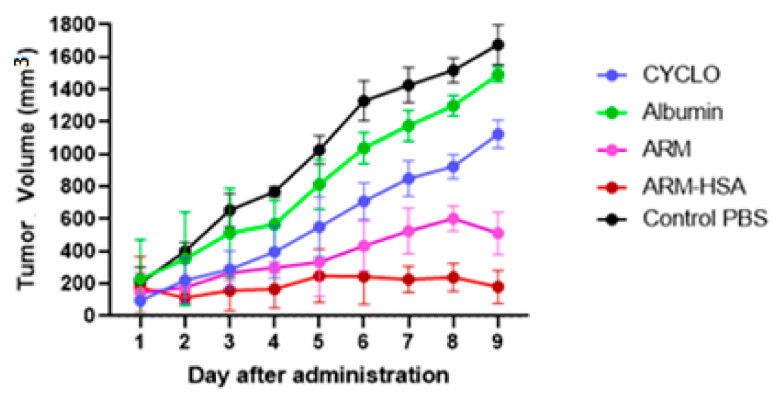
Results of tumor growth rate during 7 stages of drug injection. The highest tumor growth rate was in the PBS-receiving group. The lowest tumor growth rates were seen in the nanoparticle- and artemether-receiving groups, respectively.

Tumor mice were divided into five groups receiving cyclophosphamide, albumin, PBS, free artemether and encapsulated artemether. They received drugs in 7 steps. The results of monitoring tumor growth over 2 weeks are shown in Figure 8. According to the figure, the fastest and highest tumor growth was in the PBS- and albumin-receiving groups, whereas the nanoparticle-receiving group demonstrated the slowest tumor growth compared to others.

#### 3.4.2. ELISA Assay

**Figure 9:**
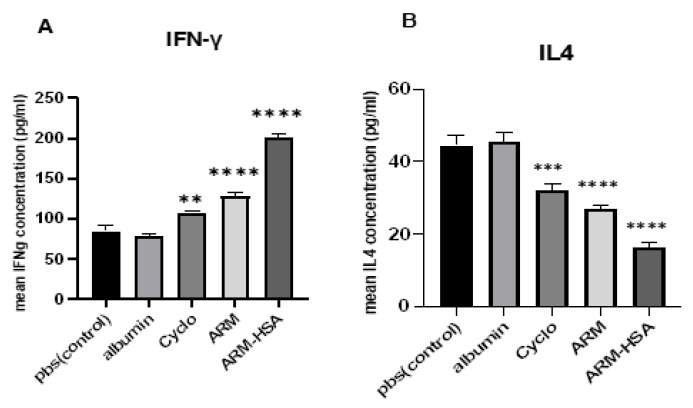
Results of IFNγ (**A**) and IL4 (**B**) secretion in five groups receiving PBS, albumin, cyclophosphamide, artemether and nanoparticles. Asterisks show the statistically significant difference between various groups. (** *p* < 0.01, *** *p* < 0.001, and **** *p* < 0.0001). The significance of data from each were compared to the control group.

To investigate changes in the secretion of IFNγ and IL4 cytokines, ELISA assay was performed. Two mice from each group were randomly selected and their splenocytes were cultured with tumor lysate for 72 h. Then, the supernatant of the samples was collected and their IFNγ and IL4 levels were determined by ELISA (Figure 9). The highest secretion of IFNγ is observed in the group receiving nanoparticles. The artemether- and cyclophosphamide-receiving groups then showed the highest cytokine secretion, respectively (Figure 9A). The results of IL4 secretion revealed that the highest level of IL4 secretion was observed in the control group receiving PBS. Afterwards, cyclophosphamide and artemether groups had the highest IL4 secretion, respectively. The lowest level of IL4 secretion was observed in the group receiving nanoparticles (Figure 9B).

#### 3.4.3. Survival of Tumor Mice

**Figure 10:**
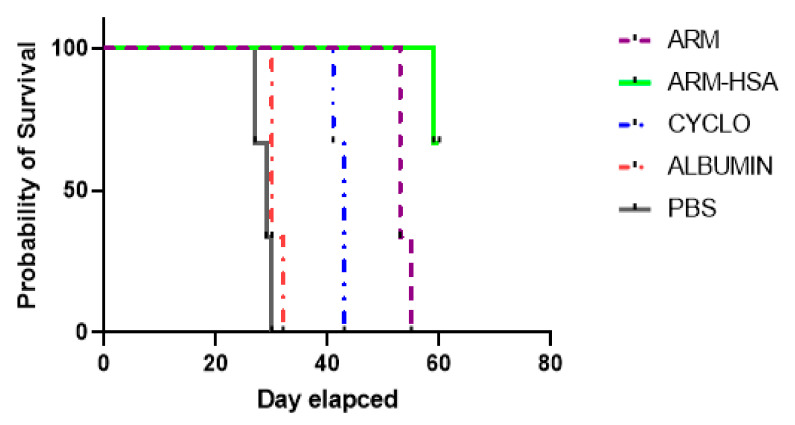
Survival chart of tumor mice receiving artemether, nanoparticle, albumin, cyclophosphamide and PBS during 60 days after treatment. Alive mice up to day 60 were found only in the nanoparticle-receiving group. Mice in other groups died before day 60.

Three mice from each group were considered for survival analysis. Figure 10 shows the survival of tumor mice up to 60 days after receiving the drug. Mice with a tumor size of 2000 mm^3^ or more were considered dead. Mice receiving Albumin and pbs survived for up to 30 days and died after the mentioned time. The only injection group in which the mice survived after 60 days was the nanoparticle receiving group.

## 4. Discussion

This study aimed to enhance the effectiveness of artemether by converting it to an encapsulated form with albumin. Hydrophobic drugs can be encapsulated by albumin. The advantages of using albumin include its non-immunologicality, non-toxicity and degradable nature. Albumin has the characteristic of preferential uptake by tumors and inflamed tissues. Defective blood vessels of tumor tissue increase the permeability of its vessels for macromolecules, while in blood vessels of healthy tissue, only small molecules can pass through the endothelial barrier. This is called the “EPR effect”, which causes the accumulation of macromolecules in the tumor tissue. The pore size of tumor microvessels can vary from 100 to 1200 nm in diameter [55,56,57]. In this study, artemether was encapsulated by Human Serum albumin (HSA). In fact, the glutaraldehyde used in this process stabilizes the amide bonds formed between the albumin so that the albumin can encapsulate the artemether [58]. It should be noted that during the encapsulation process, no bond is formed between artemether and the HSA particles, and there are weak bonds formed between artemether and albumin such as hydrogen, electrostatic and hydrophobic interactions [59,60]. In the first stage, after the production of nanoparticles, it was ascertained that the artemether and albumin structures remained intact by FTIR analysis (Figure 1). The findings demonstrated the main peaks of the artemether in the structure of the nanoparticles without any chemical changes (three characteristic peaks at 872.76 cm^−1^, 1035.17 cm^−1^ (C-O), and 1104.91 cm^−1^ (C-O-C, ether)). The main peaks of albumin were also visible in the nanoparticle structure. In addition, the comparison of this FTIR spectrum with previous articles confirmed the accuracy of the encapsulation process [61,62]. Previous studies on albumin nanoparticles found them as as spherical NPs with a smooth surface [41,42]. The morphology of the nanoparticles was examined by SEM and TEM microscopy (Figure 3). Comparing the image of irregular and scattered artemether particles with an image of regular and spherical nanoparticles, it can be concluded that artemether particles were encapsulated by albumin. One of the factors influencing the effectiveness of anti-tumor nanoparticles is their size. The EPR effect causes the accumulation of macromolecules in the tumor environment. The EPR effect directly depends on the size of macromolecules [63,64]. Small-sized drugs can diffuse in/out of the blood vessels of the tumor, while larger drugs (between 20 and 200 nm) can enter the interstitial space of the tumor without returning to the bloodstream [65]. In 2003, the results of a study conducted by Langer et al. showed that reducing the particle size from 800 to 200 nm mitigated phagocytosis by macrophages. On the other hand, it was shown that if their size is smaller than 100 nm, nanoparticles may deposit in the liver [51]. In this study, the DLS results showed that the average nanoparticle size was 171 nm, which was considered in the optimal range. Another important parameter is the charge of nanoparticles. Higher-charged nanoparticles (positive or negative), due to repulsion, reduce the likelihood of their accumulation [66]. According to Table 1, the charge of nanoparticles was—19.1 mV. A negative charge reduces the possibility of nanoparticle opsonization by macrophages and decreases the likelihood of particle aggregation with plasma proteins [67]. PDI is an indicator of the homogeneity of the particle size. In previous studies, it has been shown that the optimal PDI for albumin nanoparticles is between 0.05 and 0.2 [52]. In this study, the particle size distribution was measured at 0.132. The results of drug release (Figure 4) showed that in the citrate buffer, about 40% of artemether was released into the environment in the first 12 h. This value had a completely upward trend for up to 72 h, indicating that after this time, almost 80% of artemether had been released. In the phosphate buffer, the release rate was much lower so that in the first 20 h, less than 10% of the drug was released into the environment, which did not change significantly during 72 h. These results indicate that most drug delivery occurs in the target tumor environment and the drug release is very low before reaching the target site. Release at the target site is one of the characteristics of an ideal drug. In 2017, a study was conducted on the role of artemether in the treatment of lymphoma. It was shown that artemether could induce cell apoptosis by stopping the cell cycle through inducing the expression of cell cycle-suppressing proteins and increasing the activity of factors such as caspase-3 [68]. In this study, the effect of free artemether on the cell death of CT26 mouse colon cancer cell line was compared with the effect of encapsulated artemether using MTT assay. The MTT (3-[4,5-dimethylthiazol-2-yl]-2,5 diphenyl tetrazolium bromide) assay is based on the conversion of MTT into formazan crystals by the mitochondrial activity of living cells and is a known test to estimate cytotoxicity after various treatments [69]. According to MTT results (Figure 5), we can conclude that the effect of artemether, like many other herbal medicines, depends on concentration and time [70,71]. In 2013, a study was carried out on the effect of artemether on a human glioma cell line. MTT results showed that the highest cytotoxicity effect of artemether occurred within 48 h [72]. In the present study, the effect of free artemether was observed to have not increased significantly after 48 h, whereas the effect of the encapsulated artemether increased after 48 h, and the cell survival decreased to about 50% in 72 h. In diagrams of 48 and 72 h at all concentrations, there was a significant difference between the cytotoxic effect of free artemether and encapsulated artemether. The gradual release of artemether, the improvement of stability, and solubility due to the conversion of the free form of artemether to the nanoparticle form are some of the reasons that increase the effect of nanoparticles compared to artemether. A comparison of IC 50 results with similar articles shows that the difference between the IC 50 value of nanoparticles and free artemether is significant [45,73]. According to Figure 6, the results of the IC50 calculation show that artemether encapsulation with albumin reduces the effective dose of the drug. Annexin PI apoptosis assay was performed to investigate the mechanism of cell death. A major mechanism of action of artemisinin and its derivatives against cancer cells is the induction of apoptosis, which leads to activation of caspase-3 by elevating the expression of the Bax/BCl2 ratio and inducing the release of cytochrome c from mitochondria [20,74]. Iron in cancer cells also activates and breaks down the artemisins endoperoxide bridge, resulting in the formation of free radicals and a caspase-independent apoptotic pathway. Due to the fact that the tolerance threshold for reactive oxygen species (ROS) in cancer cells is lower than normal cells due to the lack of antioxidant proteins [75], artemether induces death in cancer cells by producing ROS. However, increasing the concentration of artemether may cause ROS to affect normal cells as well, which limits the use of high concentrations of artemether. Therefore, the use of nano-formulations that significantly reduce the effective concentration can be useful. Figure 7 shows that the rate of primary apoptosis, secondary apoptosis, and total apoptosis in the nanoparticle-treated group is significantly higher than those of the free Artemether-treated group. The number of cells that entered the primary apoptosis analysis in 24 h is more than the number of cells that entered the secondary apoptosis phase. Accordingly, it can be inferred that the cells in the secondary apoptosis group have undergone the apoptotic pathway from the beginning but have not undergone necrosis. In addition, another study using DNA diffusion assays to investigate apoptosis induction by artemisinin derivatives on human cancer cells revealed that the effect of artemisinin derivatives was exerted through the induction of apoptosis but not necrosis [76]. A possible reason for the difference between the results of the apoptosis analysis and the MTT cytotoxicity assay is that in the MTT cytotoxicity assay, cells with primary apoptosis may also be considered living cells. However, in the analysis of apoptosis, which is a quantitative method, the evaluated cells are distinguished separately into living cells, necrosis, primary apoptosis and secondary apoptosis [53] The antitumor effects of artemether have been investigated by in vivo studies in recent years [77]. In this study, in vivo studies were performed to evaluate the effect of encapsulated artemether on tumor growth and production of IFNγ and IL4 cytokines. Tumor-infected mice were treated with the drug as described in Section 2.5.2. and the drug was injected intramuscularly into the mice in seven steps. There are several methods for injecting medication including intravenously, intraperitoneally and intramuscularly. Because drugs for intravenous injection must be 100% soluble, few drugs are generally injected intravenously into mice and rats [78]. The presence of proteases and other enzymes in the peritoneum can break down the albumin structure before it reaches the tumor site [79]. Furthermore, a large number of peritoneal macrophages can lead to the phagocytosis of nanoparticles [80]. Therefore, the most appropriate method of drug injection is the intramuscular method as employed in this study. According to Figure 7, although free artemether is less effective than encapsulated Artemether, the effect of free artemether was greater than cyclophosphamide. The slope of tumor progression in the free and encapsulated artemether group was smaller than that of the cyclophosphamide receiving group. A typical response of immune system cells to tumors is the production of cytokines such as IFNγ. The shift of immune responses to Th_1_ is crucial in determining the quality of the immune responses against the tumor. Since tumors suppress antitumor responses through mechanisms such as shifting the immune response to Th_2_ via producing inhibitory cytokines such as IL4, one of the characteristics of an appropriate antitumor drug, in addition to affecting the tumor, is enhancing the antitumor response of the immune system and directing the immune system’s responses to Th_1_ [81]. On the other hand, changes in the Th_1_/Th_2_ ratio are a common feature in patients with malignancy that can be caused by defects in Th_1_ cells, activation of Th_2_ lymphocytes, or both [82]. Balancing the Th_1_ and Th_2_ responses is one of the features of an effective drug that can lead to an effective immune response against the tumor. According to our findings, the amount of IFNγ secretion in the groups receiving free and encapsulated artemether was significantly higher than that of the control group (*p* < 0.0001). There was no significant difference in IFNγ secretion in the albumin receiving group compared to the control group. The highest amount of secreted IFNγ was observed in the nanoparticle receiving group. The amount of IFNγ in the cyclophosphamide group, although significantly higher in the control group, was less significant than in the free artemether and the nanoparticles receiving group. The lowest level of IL4 secretion was seen in the NPs receiving group. Therefore, it can be concluded that the encapsulated form of Artemether compared to its free form can increase the antitumor responses by directing immune responses to Th_1_ and reducing Th_2_, which is how tumor progression can be prevented. These results also show that encapsulated artemether has a greater effect on the tumor microenvironment than free artemether and also causes more drug uptake by cells [83]. Evaluation of the survival of drug-treated mice shows the role of the drug in preventing tumor recurrence. The survival of nanoparticle-receiving tumor mice for up to day 60 also confirms the above-mentioned results.

## Figures and Tables

**Table 1:** Zeta potential, PDI and size of Albumin–artemether nanoparticles (mean ± SD).

Nanoparticles	Zeta Potential ± SD (mV)	PDI ± SD	Size ± SD (nm)
ARM-HSA NPs	−19.1 ± 0.82	0.132 ± 0.006	171.3 ± 5.88
